# Double-Cabin Galvanic Cell-Synthesizing Nanoporous, Flower-like, Pb-Containing Pd–Au Nanoparticles for Nonenzymatic Formaldehyde Sensor

**DOI:** 10.3390/molecules29122772

**Published:** 2024-06-11

**Authors:** Zhao Huang, Zhongsen Tang, Long Chao

**Affiliations:** 1 Hunan Key Laboratory of Biomedical Nanomaterials and Devices, College of Life Sciences and Chemistry, Hunan University of Technology, Zhuzhou 412007, China; huang1020@hut.edu.cn (Z.H.);; 2 Key Laboratory of Chemical Biology and Traditional Chinese Medicine Research (Ministry of Education of China), College of Chemistry and Chemical Engineering, Hunan Normal University, Changsha 410081, China

**Keywords:** Pb-containing Pd–Au nanoparticles, double-cabin galvanic cell, underpotential deposition, nonenzymatic formaldehyde sensor

## Abstract

In this work, a novel formaldehyde sensor was constructed based on nanoporous, flower-like, Pb-containing Pd–Au nanoparticles deposited on the cathode in a double-cabin galvanic cell (DCGC) with a Cu plate as the anode, a multiwalled carbon nanotube-modified glassy carbon electrode as the cathode, a 0.1 M HClO_4_ aqueous solution as the anolyte, and a 3.0 mM PdCl_2_ + 1.0 mM HAuCl_4_ + 5.0 mM Pb(ClO_4_)_2_ + 0.1 M HClO_4_ aqueous solution as the catholyte, respectively. Electrochemical studies reveal that the stripping of bulk Cu can induce underpotential deposition (UPD) of Pb during the galvanic replacement reaction (GRR) process, which affects the composition and morphology of Pb-containing Pd–Au nanoparticles. The electrocatalytic activity of Pb-containing nanoparticles toward formaldehyde oxidation was examined in an alkaline solution, and the experimental results showed that formaldehyde mainly caused direct oxidation on the surface of Pb-containing Pd–Au nanoparticles while inhibiting the formation of CO poison to a large degree. The proposed formaldehyde sensor exhibits a linear amperometric response to formaldehyde concentrations from 0.01 mM to 5.0 mM, with a sensitivity of 666 μA mM^−1^ cm^−2^, a limit of detection (LOD) of 0.89 μM at triple signal-to-noise, rapid response, high anti-interference ability, and good repeatability.

## 1. Introduction

Formaldehyde is a health-hazard chemical widely applied in many industrial processes; for instance [1], it is used as a raw material to manufacture building plates and lacquer materials, and as an intermediate for producing detergents, soaps, cosmetics, and so forth [2]. In addition, formaldehyde can also be released into the drinking water by ozonation and chlorination of some natural organic matters during recycling of industrial or domestic wastewater [3]. The extensive residue of formaldehyde in the environment causes a risk of chronic intoxication for people exposed in the pollution zone via daily ingestion of the harmful chemical, which is a serious threat to human health [4]. The International Agency for Research on Cancer has also classified formaldehyde as a human carcinogen [5]. Therefore, it is important to develop a rapid and highly sensitive method to monitor the trace concentrations of formaldehyde in potentially contaminated fields.

Up to now, various analytic methods have been explored to determine formaldehyde concentrations at trace levels; for instance, spectrophotometry [6], gas chromatography [7], and high-performance liquid chromatography [8]. However, these methods usually suffer from the drawbacks of high cost, being time consuming, and complex operation, limiting their use in monitoring trace concentrations of formaldehyde in real time. On the other hand, electrochemical sensors have the advantages of low cost, simplicity, sensitivity, and on-the-spot detection. Intensive studies have been devoted to exploiting high-performance formaldehyde electrochemical sensors [9,10,11]. For instance, Yinuo Zhang et al. reported a formaldehyde electrochemical biosensor based on the selective electrochemical oxidation of formaldehyde catalyzed by aldehyde dehydrogenases (ALDHs) on a Cu electrode, with a limit of detection (LOD) of 1.46 × 10^−15^ M [9]. Shimomura et al. developed an enzymatic biosensor based on mesoporous silica materials (FSM8.0 or P123-M) for immobilizing formaldehyde dehydrogenase (FDH), and the LOD was reduced to 1.2 μM [12]. Although the enzymatic biosensor exhibits excellent sensitivity and good selectivity toward formaldehyde detection, the inevitable shortcomings that arise from the intrinsic nature of enzymes, including chemical and thermal instability as well as a short lifetime, hinder their wide application. At present, nonenzymatic electrochemical organic small-molecule sensor-based nanomaterials have been widely studied [13,14]. Therefore, preference is given to nanomaterial-modified, non-enzymatic sensors, owing to their high electrocatalytic activity towards formaldehyde. For instance, nanostructured Pd–Pt or Pd–Au electrocatalysts have been adopted to construct nonenzymatic formaldehyde sensors [15,16]. However, there are two key problems associated with nonenzymatic formaldehyde sensors: first, low sensitivity originating from the sluggish kinetics of formaldehyde electrooxidation on a poisoned interface; and second, poor selectivity derived from the oxidation of formaldehyde and other electroactive species in the same potential range, which should be addressed before practical applications. Hence, improving the performance of electrode materials in many ways is an important idea to address the limitations on the development of non-enzymatic formaldehyde electrochemical sensors.

As is well known, multicomponent metal nanoparticles always exhibit enhanced electrocatalytic performance compared with the corresponding monometallic nanoparticles, owing to the synergistic effects between different components [17,18]. Palladium-based nanomaterials have also been widely applied in electrocatalytic oxidation and electrochemical detection of formaldehyde molecules [19,20]. Among the various palladium-based nanoparticles, Pd–Au bimetallic nanoparticles can strongly absorb light and efficiently enhance the conversion of several organic reactions under visible light irradiation at ambient temperatures [21]. In addition, effectively controlling the morphology of nanoparticles can further improve their performance, as the electrochemical reaction pathways are highly sensitive to the surface atomic arrangement exposed on special facets. A variety of Pd–Au nanoparticles with shapes such as hexoctahedral nanocrystals [22], porous flower [23], core–shell shape [24], etc., have been prepared and investigated. The relatively large surface areas and highly active facets on their surfaces endow the prepared nanoparticles with enhanced catalytic efficiencies.

To date, various strategies, for example, wet-chemical synthesis, self-assembly, electrodeposition, the galvanic replacement reaction (GRR), etc., have been explored to prepare metal micro/nanostructures of specific morphologies [25,26,27,28]. Among these, the eco-friendly GRR receives particular attention because of its tunability, economy, and simplicity. Typically, the GRR-based synthesis involves the replacement of relatively active metal templates (for example, Cu and Ag nanostructures) by target noble metal ions, as driven by the difference in electrochemical potential (ECP) between metal templates (lower ECP) and noble metal ions (higher ECP) [29]. The conventional GRR synthetic process always works in a manner such that the anode and cathode coexist in a one-pot solution, in which the metal templates’ impurities and the time-consuming, post-purifying step are inevitable. However, physically separating the cathodic and anodic processes of the GRR in a double cabin galvanic cell (DCGC) can overcome the above-mentioned disadvantages for synthesizing nanomaterials; moreover, the substrate-supported nanostructured materials synthesized in the DCGC can be used directly as prepared, avoiding the post-purifying step, but such a convenient DCGC step based on the GRR principle is rarely attempted in fabricating highly efficient nanoelectrocatalysts with controllable morphologies and compositions.

Here, a novel formaldehyde sensor is designed and fabricated using the nanoporous, flower-like, Pb-containing Pd–Au nanoparticles synthesized in the DCGC as the electrochemical sensing element. The developed Pb-containing Pd–Au nanoparticles exhibit remarkable catalytic activity toward formaldehyde electrooxidation and efficiently suppress the formation of CO poison, which is beneficial for constructing a high-performance formaldehyde sensor.

## 2. Results and Discussion

### 2.1. Fabrication and Characterization of Pb-Containing Pd–Au Nanoparticles

**Figure 1:**
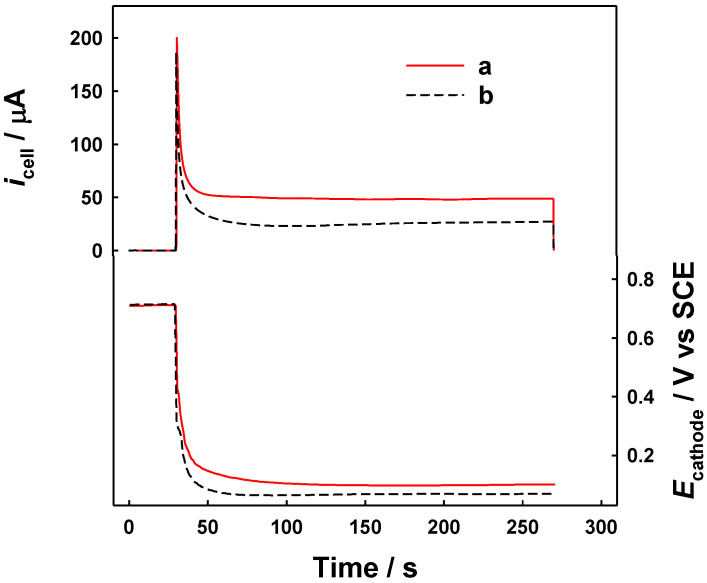
Simultaneous responses of *i*_cell_, and *E*_cathode_ in the DCGC with a Cu plate as the anode, the MWCNTs/GCE as the cathode, and 0.1 M HClO_4_ as anolyte (stationary), while the catholytes (stationary) were 3.0 mM PdCl_2_ + 1.0 mM HAuCl_4_ +5.0 mM Pb(ClO_4_)_2_ + 0.1 M HClO_4_ aqueous solution (a, for deposition of Pb-Pd_3_Au_1_) and 3.0 mM PdCl_2_ + 1.0 mM HAuCl_4_ + 0.1 M HClO_4_ aqueous solution (b, for deposition of Pd_3_Au_1_), respectively. Short-circuit discharging of the DCGC (connecting the anode and cathode by a conducting Cu wire) was from 30 s to 270 s.

Figure 1 shows the discharging current (*i*_cell_) and potential of the cathode during the deposition of metallic nanoparticles on the MWCNTs/GCE (cathode) based on the GRR principle. After connecting the anode and cathode by a conducting Cu wire in the DCGC, the ECP difference between the anode part (ca. 0.01 V for Cu plate in 0.1 M HClO_4_) and the cathode part (ca. 0.78 V for MWCNTs/GCE in a noble metal mixed salts solution) derived the occurrence of GRR, resulting in the stripping of the bulk Cu (Cu_bulk_) anode and deposition of noble metal nanoparticles onto the MWCNTs/GCE cathode. During deposition of Pd_3_Au_1_ from the Pb(ClO_4_)_2_-free 3.0 mM PdCl_2_ + 1.0 mM HAuCl_4_ + 0.1 M HClO_4_ catholyte (Figure 1(b)), the steady-state i_cell_ and potential of the MWCNTs/GCE (*E*_MWCNTs/GCE_) were 27 μA and 0.068 V, respectively. However, for deposition of Pb-Pd_3_Au_1_ from the 3.0 mM PdCl_2_ + 1.0 mM HAuCl_4_ + 5.0 mM Pb(ClO_4_)_2_ + 0.1 M HClO_4_ catholyte (Figure 1(a)), the steady-state i_cell_ and E_MWCNTs/GCE_ were 48 μA and 0.098 V, both of which are lager than those for deposition of Pd_3_Au_1_, perhaps implying the enhanced deposition of Pd and Au in the presence of Pb(ClO_4_)_2_.

**Figure 2:**
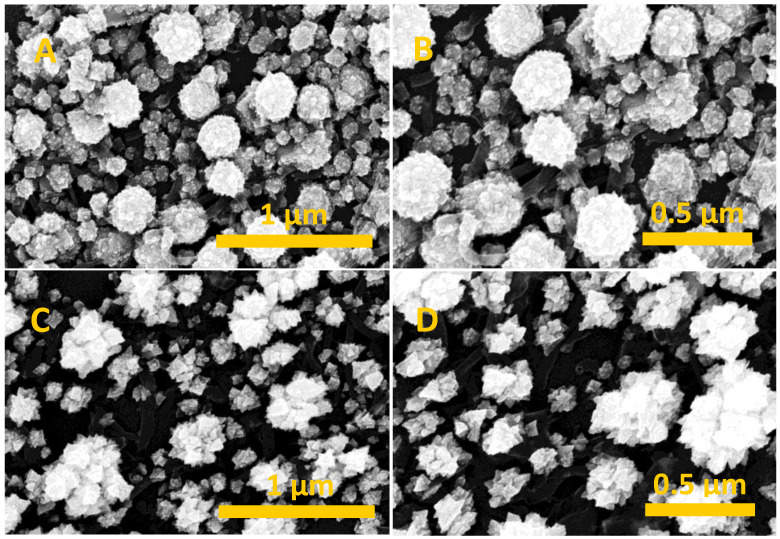
SEM of Pd_3_Au_1_/MWCNTs/GCE (**A**,**B**) and Pb-Pd_3_Au_1_/MWCNTs/GCE (**C**,**D**).

**Figure 3:**
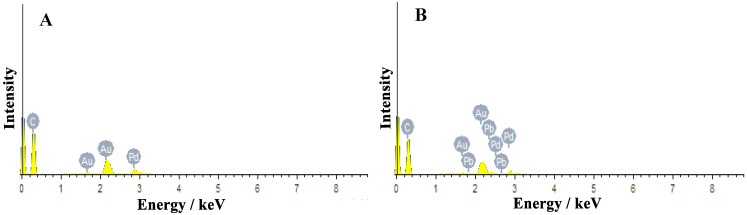
EDS spectra of Pd_3_Au_1_/MWCNTs/GCE (**A**) and Pb-Pd_3_Au_1_/MWCNTs/GCE (**B**).

Figure 2 shows SEM images of the MWCNTs/GCE-supported Pd_3_Au_1_ and Pb-Pd_3_Au_1_ metallic nanoparticles, and SEM image of the MWCNTs/GCE is also shown in Figure S1. Spindly MWCNTs can be observed uniformly dispersed on the GCE before the modification of the nanoparticles. For the Pd_3_Au_1_ nanoparticles (panels A and B), walnut kernel-like nanoparticles with rough surfaces were distributed on the MWCNTs, with diameters ranging from 60 nm to 230 nm. However, for Pb-Pd_3_Au_1_ nanoparticles (panels C and D), nanoporous flowers of 70~500 nm in diameter were formed on the MWCNTs. In fact, close inspection of the larger Pb-Pd_3_Au_1_ nanoparticles shows that flower-like nanoparticles are built up by many smaller nanoparticles with non-isotropic structures, and nanochannels or nanovoids between the smaller nanoparticles are present on the surface. EDS characterization indicated that Pd and Au were deposited on the surfaces of the MWCNTs (Figure 3), and the atomic percentages of Pd and Au for the Pd_3_Au_1_ nanoparticles were determined to be 48% and 52% (Figure 3A), respectively, which was un-proportional to that of the feeding solution (mole ration for Pd/Au of 3:1 in the catholyte). Because of the higher ECP of Au versus that of Pd, the reaction kinetics of reducing AuCl_4_^−^ are expected to be much faster than that of reducing PdCl_2_, thus leading to the deposition of more Au in the Pd_3_Au_1_ nanoparticles, even at a high Pd/Au mole ratio in the catholyte. In addition, the component of Pb was detected in Pb-Pd_3_Au_1_ nanoparticles (Figure 3B), and the atomic percentage of Pd, Au, and Pb were determined to be 35%, 56%, and 9.0%, respectively, which indicates the occurrence of cathodic deposition of Pb from the Pb(ClO_4_)_2_-containing catholyte during the GRR deposition of Pb-Pd_3_Au_1_ nanoparticles, though bulk Pb (Pb_bulk_) is more active than Cu_bulk_ (anode here). Here, it is interesting that deposition of Pb not only induces the formation of nanoporous flowers but also changes the content of Pd and Au in Pb-Pd_3_Au_1_ nanoparticles (35% Pd and 56% Au in Pb-Pd_3_Au_1_ nanoparticles versus 48% Pd and 52% Au in Pd_3_Au_1_ nanoparticles). Virtually, previous studies have also reported that the additives of transition metal salts (for instance CuCl_2_, AgNO_3_) can affect the growth of nanostructured noble metal materials [30,31].

### 2.2. Electrochemical Study of the Evolution of Cathodic Deposition of Pb during the GRR Process

**Figure 4:**
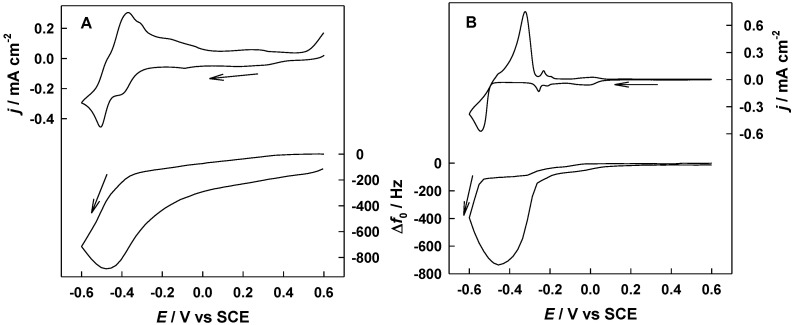
Simultaneous responses of current and Δ*f*_0_ on Pd_pla_/Au (**A**) or Au (**B**) during potential cycling in 3.0 mM Pb(ClO_4_)_2_ + 0.1 M HClO_4_ solution. The arrow indicates the scanning direction. Scan rate: 20 mV s^−1^.

It is important to study the evolution of cathodic deposition of Pb in the DCGC with a Cu_bulk_ anode, since the ECP of Pb_bulk_ is lower than that of Cu_bulk_, thus the stripping of Cu_bulk_-induced deposition of Pb_bulk_ is thermodynamically impossible during GRR deposition of Pb-Pd_3_Au_1_. Here, we conducted the cyclic voltammetry (CV) experiments to examine the electrochemical behaviors of Pb on electroplated Pd-modified Au (Pd_pla_/Au, as shown in Figure S2) or Au electrodes via EQCM method, as shown in Figure 4. For the Pd_pla_/Au electrode, the decrease of Δ*f*_0_ started at 0.46 V, corresponding to the occurrence of underpotential deposition (UPD) of Pb onto Pd_pla_/Au [32], and the UPD of Pb (Pb_UPD_) suppressed hydrogen adsorption/desorption onto Pd_pla_/Au, to a large degree, in the potential range of 0~−0.22 V. The UPD of Pb ended at −0.45 V where the deposition/stripping of Pb_bulk_ occurred, as reflected by the great decrease/increase of Δ*f*_0_ at −0.45 V. For the Au electrode, the decrease of Δ*f*_0_ started at 0.06 V, also corresponding to the occurrence of UPD of Pb on Au [33], and two pairs of distinct UPD peaks at 0.01/−0.01 V and −0.23/−0.25 V were observed. The deposition/stripping of Pb_bulk_ occurred at ca. −0.45 V, resulting in a great decrease/increase of Δ*f*_0_ at −0.45 V. From the CV experimental results, it is obvious that deposition/stripping of Pb_UPD_ occurs at more positive potentials versus that of Pb_bulk_, which means that Pb^2+^/Pb_UPD_ has a higher ECP than Pb^2+^/Pb_bulk_. Furthermore, both the ECP of Pb^2+^/Pb_UPD_ on Pd (exclusively for 0.46~0.01 V) and ECP of Pb^2+^/Pb_UPD_ on Au (exclusively for 0.06~0.01 V) are even higher than that of Cu^2+^/Cu_bulk_ (0.01 V here, Figure S3), implying that the stripping of Cu_bulk_-induced deposition of Pb_UPD_ onto Pd or Au is thermodynamically possible.

Since the steady-state *E*_MWCNTs/GCE_ for deposition of Pb-Pd_3_Au_1_ was 0.098 V (Figure 1(a)), matching well with the potential range for UPD of Pb on Pd (−0.45~0.46 V), but higher than that for UPD of Pb on Au (−0.45~0.06 V), the occurrence of UPD of Pb on Pd during GRR deposition of Pb-Pd_3_Au_1_ is reasonable. Hence, the ECP difference between Pb^2+^/Pb_UPD_ on Pd and Cu^2+^/Cu_bulk_ can account for the cathodic deposition of Pb in the DCGC with a Cu_bulk_ anode. Such a UPD effect of Pb can induce the growth of Pd and Au nanoparticles to form nanoporous, flower-like Pb-containing Pd_3_Au_1_ nanostructures, the catalytic performance of which may also be improved via the synergistic interaction between Pd, Au, and Pb.

### 2.3. Electrochemical Behaviors and Electrocatalytic Responses to Formaldehyde on Modified Electrodes

**Figure 5:**
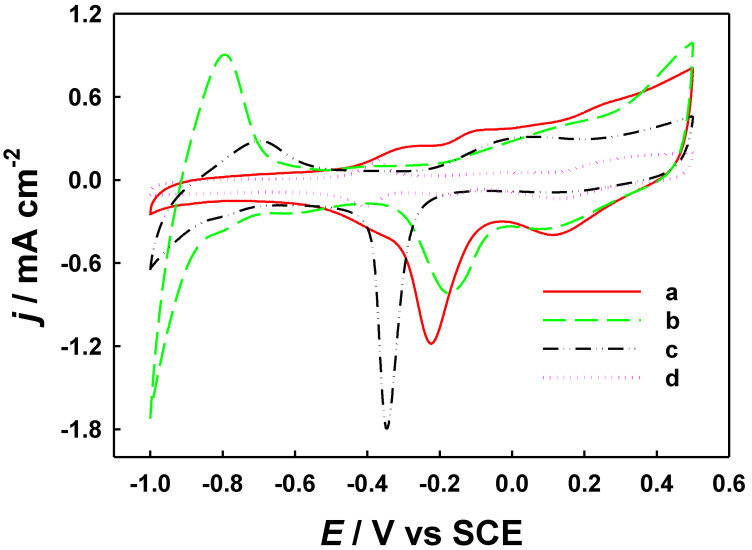
CV curves of Pb-Pd_3_Au_1_/MWCNTs/GCE (a), Pd_3_Au_1_/MWCNTs/GCE (b), Pb-Pd/MWCNTs/GCE (c), and Pb-Au/MWCNTs/GCE (d) in 0.1 M KOH aqueous solution. Scan rate: 50 mV·s^−1^.

Figure 5 shows the cyclic voltammograms (CVs) of several modified electrodes in 0.1 M KOH aqueous solution. The reduction peak of Pd oxides (P_c-Pd_) appeared at −0.34 V on Pb-Pd/MWCNTs/GCE, accompanied by the peaks of hydrogen adsorption/desorption at −0.66~−1.0 V. The reduction peak of Au oxides (P_c-Au_) was observed at 0.13 V on Pb-Au/MWCNTs/GCE. For Pb-Pd_3_Au_1_/MWCNTs/GCE, the potential of P_c-Au_ is consistent with that on Pb-Au/MWCNTs/GCE; however, the P_c-Pd_ (−0.22 V) positively shifted by 120 mV versus that on Pb-Pd/MWCNTs/GCE (−0.34 V); similar potential shifting of P_c-Pd_ was also observed in the electrochemical behaviors of Pd–Au alloy nanoparticles [22]. In addition, compared with Pd_3_Au_1_/MWCNTs/GCE, Pb-Pd_3_Au_1_/MWCNTs/GCE did not produce distinct peaks of hydrogen adsorption/desorption, indicating that the electrochemical behaviors of Pb-Pd_3_Au_1_/MWCNTs/GCE depended on the unique morphology and the atomic arrangement of Pd, Au, and Pb on the surface.

**Figure 6:**
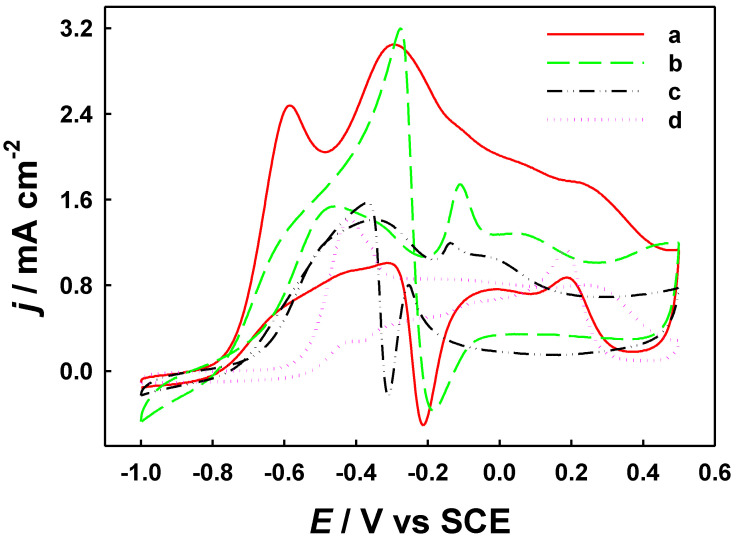
CV curves of Pb-Pd_3_Au_1_/MWCNTs/GCE (a), Pd_3_Au_1_/MWCNTs/GCE (b), Pb-Pd/MWCNTs/GCE (c), and Pb-Au/MWCNTs/GCE (d) in 10 mM HCHO + 0.1 M KOH aqueous solution. Scan rate: 50 mV·s^−1^.

For the electrooxidation of formaldehyde (Figure 6), during the forward scan, two oxidation peaks appeared at −0.58 and −0.28 V on Pb-Pd_3_Au_1_/MWCNTs/GCE, and the oxidation peak potentials were lower than those on Pd_3_Au_1_/MWCNTs/GCE (−0.46 and −0.11 V) and Pb-Pd/MWCNTs/GCE (−0.34 and −0.14 V). No peaks for the electrooxidation of formaldehyde appeared on MWCNTs/GCE. The maximum oxidation peak current was reached on Pb-Pd_3_Au_1_/MWCNTs/GCE (3.06 mA cm^−2^, −0.28 V), indicating the highest catalytic activity of Pb-Pd_3_Au_1_/MWCNTs/GCE toward formaldehyde electrooxidation. During the backward scan, an oxidation peak appeared at 0.19 V on Pb-Pd_3_Au_1_/MWCNTs/GCE, similar to that observed on Pb-Au/MWCNTs/GCE. After the reduction of Pd oxides at more negative potential, another oxidation peak appeared at ca. −0.3 V on Pb-Pd_3_Au_1_/MWCNTs/GCE, corresponding to the oxidation of the accumulated CO poisonous intermediate generated through oxidation of formaldehyde during the forward scan [34], and the oxidation peak current (1.01 mA cm^−2^) was lower than that on Pd_3_Au_1_/MWCNTs/GCE (3.19 mA cm^−2^, −0.27 V) and Pb-Pd/MWCNTs/GCE (1.56 mA cm^−2^, −0.36 V). Previous studies reported that the oxidation mechanism of formaldehyde on palladium follows a dual pathway [24,35], including a direct oxidation pathway and an indirect oxidation pathway. Formaldehyde is directly oxidated to CO_2_ at a low potential in the former, but indirectly oxidated to CO_2_ via the chemisorbed CO intermediate at a high potential in the latter. The presence of oxidation peaks in the forward and backward scans indicates direct and indirect pathways of formaldehyde oxidation in the overall process. Here, the lower oxidation peak potentials (−0.58 and −0.28 V) and the maximum oxidation peak current (3.06 mA cm^−2^, −0.28 V) on Pb-Pd_3_Au_1_/MWCNTs/GCE during the forward scan, as well as the lower oxidation peak current (1.01 mA cm^−2^, −0.3 V) during the backward scan, prove that Pb-Pd_3_Au_1_/MWCNTs/GCE shows remarkably improved electrocatalytic activity toward formaldehyde oxidation, mainly through a direct oxidation pathway (suppressing the formation of CO poison) among the examined electrocatalysts. The enhanced performance on Pb-Pd_3_Au_1_/MWCNTs/GCE can be attributed to nanoporous flower-like Pb-Pd_3_Au_1_ nanoparticles with highly active sites in greater quantity on the large areal surface, firstly; secondly, the favorable electronic effect and bifunctional effect between Pd, Au, and Pb atoms facilitate the direct oxidation of formaldehyde [36]; thirdly, facile mass transfer for formaldehyde and the oxidized products on the nanoporous surface of Pb-Pd_3_Au_1_ nanoparticles.

### 2.4. Amperometric Sensing of Formaldehyde

To construct a formaldehyde amperometric sensor based on the Pb-containing Pd–Au nanoparticles, the effect of sensing conditions, such as applied potential and the atomic ratio of Pd to Au in the nanoparticles, were first optimized. Figure S4 shows the effects of the applied potential on the amperometric response of 50 μM HCHO for Pb-Pd_3_Au_1_/MWCNTs/GCE in 0.1 M KOH aqueous solution. From Figure S4, the maximum amperometric response was achieved under −0.27 V; thus, we chose −0.27 V as the applied potential to further optimize the atomic ratio of Pd to Au in the nanoparticles by adjusting the molar concentration ratio of PdCl_2_ to HAuCl_4_ in the catholyte, as shown in Figure S5. When the molar concentration ratio of PdCl_2_/HAuCl_4_ was 3:1, the prepared Pb-Pd_3_Au_1_/MWCNTs/GCE gave the largest amperometric response toward formaldehyde electrooxidation. In addition, the amperometric response of Pb-Pd_3_Au_1_/MWCNTs/GCE was also superior to that of Pd_3_Au_1_/MWCNTs/GCE and Pb-Pd/MWCNTs/GCE, in accordance with the results from the above electrocatalytic experiments (Figure 6).

**Figure 7:**
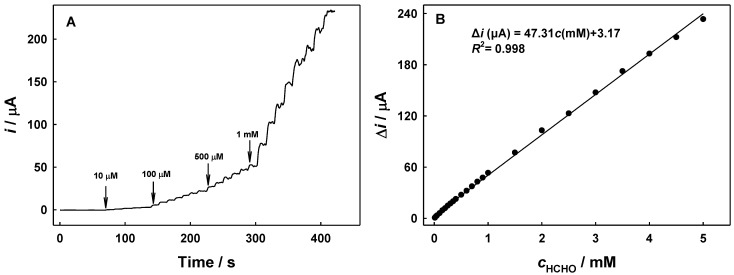
Amperometric response to successive addition of HCHO into the stirred 0.1 M KOH aqueous solution (**A**) and the calibration curve (**B**) at Pb-Pd_3_Au_1_/MWCNTs/GCE. Applied potential: −0.27 V.

For the electrochemical determination of formaldehyde, the optimized Pb-Pd_3_Au_1_/MWCNTs/GCE electrode was employed as a working electrode, and amperometric measurements were performed at a constant applied potential of −0.27 V. Figure 7A shows the amperometric response of Pb-Pd_3_Au_1_/MWCNTs/GCE toward successive addition of formaldehyde into a stirred 0.1 M KOH aqueous solution. After adding formaldehyde, the steady-state signal was reached within 5 s, indicating the rapid response of Pb-Pd_3_Au_1_/MWCNTs/GCE towards the change in the concentration of formaldehyde. Figure 7B shows the calibration curve of the amperometric response. The sensor exhibits a linear detection range (LDR) to formaldehyde concentration from 0.01 mM to 5.0 mM, and the linear regression equation is Δ*i*(μA) = 47.31*C*(mM) + 3.17 (*R*^2^ = 0.998), with a sensitivity of 666 μA mM^−1^ cm^−2^. The LOD was estimated to be 0.89 μM at the triple signal-to-noise ratio. Comparison with the previous reported non-enzymatic formaldehyde sensors, as listed in Table 1, the sensor proposed here shows good performance in terms of the suitably wide LDR and the lower LOD.

### 2.5. Interference Tests, Repeatability, and Stability of the Sensor

**Figure 8:**
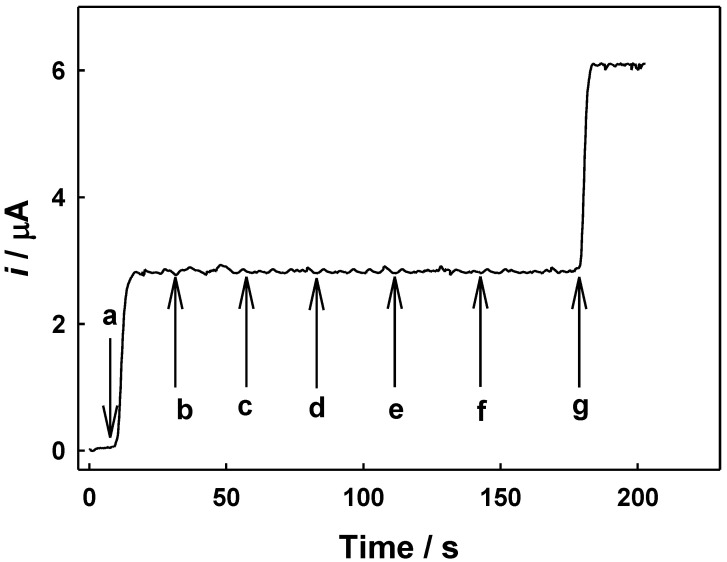
Amperometric response of Pb-Pd_3_Au_1_/MWCNTs/GCE to successive addition of 50 μM HCHO (a), 0.5 mM CH_3_OH (b), 0.5 mM C_2_H_5_OH (c), 0.5 mM HCOOH (d), 0.5 mM C_3_H_8_O (e), 0.5 mM C_3_H_6_O (f), and 50 μM HCHO (g) into the stirred 0.1 M KOH aqueous solution. Applied potential: −0.27 V.

To evaluate the anti-interference ability of the sensor in the accurate determination of formaldehyde, the effects of potentially interfering species, including methanol, ethanol, formic acid, n-propanol, and acetone, were tested. Figure 8 shows the amperometric response of Pb-Pd_3_Au_1_/MWCNTs/GCE to successive addition of 50 μM HCHO (a), 0.5 mM CH_3_OH (b), 0.5 mM C_2_H_5_OH (c), 0.5 mM HCOOH (d), 0.5 mM C_3_H_8_O (e), 0.5 mM C_3_H_6_O (f), and 50 μM HCHO (g) into the stirred 0.1 M KOH aqueous solution at a potential of −0.27 V. A fast current response occurred after adding formaldehyde; however, adding methanol, ethanol, formic acid, n-propanol, and acetone did not cause the obvious current changes, indicating that the tested species showed almost no interference to formaldehyde detection. These results confirmed the highly selective response of the Pb-Pd_3_Au_1_/MWCNTs/GCE toward formaldehyde.

Repeatability of the sensor was examined by measuring the amperometric responses of Pb-Pd_3_Au_1_/MWCNTs/GCE toward 15 successive detections of 50 μM formaldehyde in 0.1 M KOH aqueous solution, and the current response for each detection was closed to 2.6 μA, yielding a relative standard deviation (RSD) of 5.3%, indicating a good repeatability. In addition, the fabrication repeatability was also examined via measuring the amperometric responses of five Pb-Pd_3_Au_1_/MWCNTs/GCE electrodes prepared independently by the same procedure; the RSD is 4.6% for the 50 μM formaldehyde amperometric responses, demonstrating the reliability of the fabrication procedure.

Long-term stability of the sensor was investigated by regularly measuring the amperometric responses of Pb-Pd_3_Au_1_/MWCNTs/GCE (placed in the refrigerator under 4 °C) toward 50 μM formaldehyde in two weeks. The current response was 97% of its initial response after two days, and 94% of its initial response after one week. With the experiment prolonged, a further decrease in the response current was observed, retaining about 90% of its initial response after two weeks, which demonstrates that the Pb-Pd_3_Au_1_/MWCNTs/GCE has a satisfactory stability for electrochemical detection of formaldehyde (as shown in Figure S6).

### 2.6. Real-Sample Analysis

To illustrate the feasibility of the sensor in a real-sample analysis, Pb-Pd_3_Au_1_/MWCNTs/GCE was used to detect formaldehyde in tap water and lake water, respectively. The standard addition method was employed here, and the results are summarized in Table 2. The recoveries were in the range of 94.7–108.1%, and the RSD ranged from 3.4% to 5.5%, indicating that the sensor can be successfully applied for the practical electrochemical detection of formaldehyde in real samples.

## 3. Materials and Methods

### 3.1. Instrumentation and Reagents

All electrochemical experiments were conducted on an Autolab PGSTA 30 electrochemical workstation (Eco Chemie BV, Brunssum, The Netherlands) with the GPES 4.9 software. A conventional three-electrode electrolytic cell was used. A glassy carbon electrode (GCE) with a 3.0 mm diameter served as the working electrode (WE), KCl-saturated calomel electrode (SCE) with a salt bridge served as the reference electrode (RE), and a carbon rod electrode served as the counter electrode. All potentials herein are referenced to SCE. A research quartz crystal microbalance (QCM) (Maxtek, Ontario, CA, USA) was used for electrochemical QCM (EQCM) experiments, and 9 MHz QCM Au electrodes (0.29 cm^2^) of keyhole configuration (Beijing Chenjing Electronic Co., Beijing, China) were used. The mass change on the electrode was calculated according to the Sauerbrey equation, ∆*f*_0_ = −2.264 × 10^−6^*f*_0g_^2^∆*m*/*A*, where ∆*f*_0_ in Hz is the shift of the resonant frequency, ∆*m* in g is the mass change of the electrode, *f*_0g_ in Hz is the fundamental frequency in air, and *A* in cm^2^ is the piezoelectrically active area [41]. SEM characterization was performed on a Hitachi S-4800 high-resolution scanning electron microscope (Tokyo, Japan). Energy-dispersive X-ray spectroscopy (EDS, Oxford, UK) was used for elemental analysis of the electrocatalyst.

For the experiments conducted in the DCGC, the external-loading-free discharging current of the galvanic cell (*i*_cell_) was dynamically monitored with the electrochemical noise (ECN) module of the Autolab PGSTA 30 electrochemical workstation [42] (Tokyo, Japan). Before the experiments, the veracity of the ECN device was checked in advance by examining the discharging curves of a 1.5 V dry battery at an external loading of 100 kΩ, yielding a *V*_cell_ value of 1.501 V and an *i*_cell_ value of 15.01 µA, respectively, in accordance with Ohm’s law. A high-resistance (*R* ≥ 10^12^ Ω) potentiometer attached to the pH meter (Leici, Shanghai, China) was used to dynamically monitor the potential of the anode or cathode (vs SCE). All experiments were performed at room temperature (26 ± 2 °C) under air atmosphere.

HCHO, *N*,*N*-dimethylformamide (DMF), HAuCl_4_, and PdCl_2_ were purchased from Tianjin Chemical Reagents Station (Tianjin, China). Pb(ClO_4_)_2_, high-purity Cu plates (99.999%, 0.64 cm^2^) were purchased from Alfa Aesar company (Haverhill, MA, USA). Multiwalled carbon nanotubes (MWCNTs, 60 nm outer diameter and 40 nm inner diameter, on average) were purchased from Chengdu Organic Chemicals Co. Ltd. (Chengdu, China) and purified in concentrated acids before use [43]. All chemicals were of analytical grade or better quality, and all the solutions were prepared using Milli-Q ultrapure water (Millipore, Billerica, MA, USA, >18 MΩ cm).

### 3.2. DCGC Synthesis of Pb-Containing Pd–Au Nanoparticles on MWCNTs/GCE

The GCE was polished with 1.0 and 0.05 μm alumina slurry sequentially, and then washed ultrasonically in water and ethanol for 5 min, respectively. Then, the GCE was subjected to potential cycling (−0.3~1.0 V, 30 mV s^−1^) in 0.2 M aqueous HClO_4_ for sufficient cycles to obtain reproducible CV curves. The treated GCE gave a peak-to-peak separation of ca. 70 mV in the CV experiment of 2 mM K_4_Fe(CN)_6_ + 0.1 M Na_2_SO_4_ solution (−0.1~0.5 V, 50 mV s^−1^), indicating a well-cleaned electrode surface. 1 mg mL^−1^ MWCNTs dispersed in DMF were prepared and sonicated for 15 min, then 5 μL of the MWCNTs dispersion was cast onto the GCE (MWCNTs/GCE) and air-dried. 

**Scheme 1:**
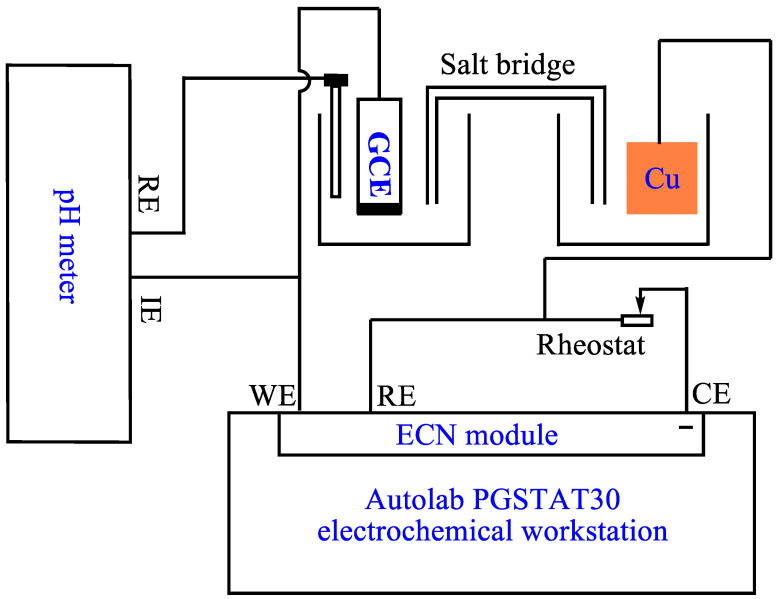
Schematic diagram of the total experimental setup. GCE: glassy carbon electrode, IE: indicator electrode.

**Scheme 2:**
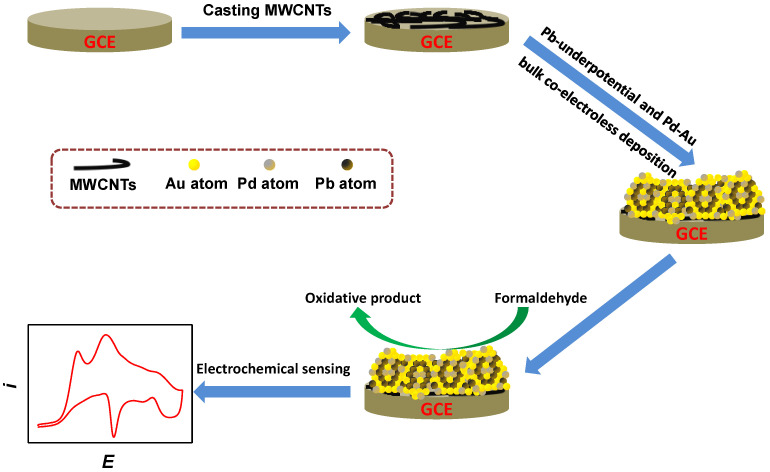
Schematic illustration of the co-electroless deposition of Pb-containing Pd–Au nanoparticles onto MWCNTs/GCE, and the electrochemical sensing of the modified electrode toward formaldehyde.

For the DCGC synthesis of Pb-containing Pd–Au nanoparticles on MWCNTs/GCE based on the GRR principle, a Cu plate served as the anode, MWCNTs/GCE served as the cathode, the anolyte was 0.1 M HClO_4_ aqueous solution, and the catholyte was 3.0 mM PdCl_2_ + 1.0 mM HAuCl_4_ +5.0 mM Pb(ClO_4_)_2_ + 0.1 M HClO_4_ aqueous solution. A salt bridge (a home-made U-tube filled with saturated KCl solution) was used to connect the anolyte and catholyte. Scheme 1 and Scheme 2 show the schematic diagram of the total experimental setup and the co-electroless deposition of Pb-containing Pd–Au nanoparticles onto MWCNTs/GCE, respectively. Figure S7 shows the physical picture display of the formaldehyde sensor with the three-electrode system. After switching on the DCGC, an ECN and pH meter were used to monitor the *i*_cell_ and the potential of MWCNTs/GCE (vs. SCE), respectively. The total discharging time in the DCGC was 240 s. The as-prepared modified electrode was denoted as Pb-Pd_3_Au_1_/MWCNTs/GCE. Varying the molar concentration ratio of PdCl_2_ to HAuCl_4_ in the catholyte can fabricate modified electrodes loaded with different atomic ratios of Pd to Au. For comparison, we also fabricated other modified electrodes in a similar procedure just by changing the catholyte into 3.0 mM PdCl_2_ +5.0 mM Pb(ClO_4_)_2_ + 0.1 M HClO_4_, 3.0 mM HAuCl_4_ +5.0 mM Pb(ClO_4_)_2_ + 0.1 M HClO_4_ and 3.0 mM PdCl_2_ + 1.0 mM HAuCl_4_ + 0.1 M HClO_4_ aqueous solution, respectively, and the corresponding modified electrodes were denoted as Pb-Pd/MWCNTs/GCE, Pb-Au/MWCNTs/GCE and Pd_3_Au_1_MWCNTs/GCE, respectively.

### 3.3. Electrochemical Characterization of the Modified Electrodes and Nonenzymatic Formaldehyde Sensor

The modified electrodes were characterized by CV in 0.1 M KOH (50 mV·s^−1^, −1.0–0.5 V) aqueous solution. The nonenzymatic formaldehyde detection was conducted potentiostatically at a fixed potential in 0.1 M KOH aqueous solution. All of the solutions were deoxygenated by bubbling high-purity N_2_ for at least 10 min prior to each measurement. The modified electrodes were kept at 4 °C in a refrigerator when not use.

## 4. Conclusions

In summary, nanoporous, flower-like, Pb-containing Pd–Au nanoparticles were deposited onto the MWCNTs/GCE cathode in DCGC with a Cu-plate anode. The presence of Pb^2+^ in the catholyte should be responsible for the morphology evolution of the Pb-containing Pd–Au nanoparticles via a UPD effect. The fabricated Pb-containing Pd–Au nanoparticles exhibit high catalytic activity toward formaldehyde electrooxidation, mainly via a direct oxidation pathway, and an optimized modified electrode (Pb-Pd_3_Au_1_/MWCNTs/GCE) was employed to fabricated a formaldehyde sensor with high sensitivity, anti-interference ability, and stability. The procedure for nanomaterial fabrication presented here should have great potential in synthesizing high-performance nanocatalysts for electrocatalysis and electroanalysis applications.

## Figures and Tables

**Table 1:** Comparison of different modified electrodes for formaldehyde electrochemical sensing.

Modified Electrode	Linear Range (mM)	LOD (μM)	References
Au@PPy composites-Glassy carbon	0.4−2.4	20	[37]
Ni-Pd/GCE	0.01–1.0	5.4	[38]
Pd-Cu-SBA-16/CPE	1.79–121.86	16	[20]
Pd_2_Pt_1_ PPy/N-rGONC/GCE	0.01−0.9	27	[15]
Ni-GCE	0.01−1.0	6	[39]
Cr-Pdene/C	0.001–0.005	1.0	[18]
CILE/Au/Pd	0.015–1.4, 1.4–56.7	3.0	[16]
nanoPd@LIG	0.01–4.0	6.4	[40]
Pb-Pd_3_Au_1_/MWCNTs/GCE	0.01–5.0	0.89	This work

Note: PPy: polypyrrole; SBA-16: Santa Barbara Amorphous no. 16; Cr-Pdene: a 2D Cr-doped Pd metallene; nanoPd@LIG: nano-palladium-grafted, laser-induced graphene; CILE: carbon ionic liquid electrode.

**Table 2:** Analysis results of formaldehyde in real samples.

Sample	Added (μM)	Found (μM)	Recovery (%)	RSD (%)
	100	102.8	102.8	5.4
Tap Water	200	201.1	100.6	3.9
	400	402.9	100.7	3.4
	100	108.1	108.1	5.5
Lake Water	200	189.4	94.7	4.1
	400	388.3	97.1	3.5

## Data Availability

Data are contained within the article and Supplementary Materials.

## Supplementary Materials

The following supporting information can be downloaded at: https://www.mdpi.com/article/10.3390/molecules29122772/s1. Figure S1. SEM images of MWCNTs/GCE. Figure S2. CV curves at Pd_pla_/Au and bare Au electrodes in 0.1 M H_2_SO_4_ aqueous solution. Figure S3. CV curves at Cu plate in 0.1 M HClO_4_ aqueous solution. Figure S4. Effects of the applied potential on the amperometric response of 50 μM HCHO for Pb-Pd_3_Au_1_/MWCNTs/GCE in 0.1 M KOH aqueous solution. Figure S5. Amperometric response to HCHO at Pb-Pd_3_Au_0.5_/MWCNTs/GCE (a), Pb-Pd_3_Au_1_/MWCNTs/GCE (b), Pb-Pd_3_Au_3_/MWCNTs/GCE (c), Pb-Pd_3_Au_5_/MWCNTs/GCE (d), Pd_3_Au_1_/MWCNTs/GCE (e), Pb-Pd/MWCNTs/GCE (f) upon successive addition of 50 μM HCHO into the stirred 0.1 M KOH aqueous solution. Applied potential: −0.27 V. Figure S6. Long-term stability of Pb-Pd_3_Au_1_/MWCNTs/GCE with repeated detection of 50 μM formaldehyde by potentiostatic method for two weeks. Applied potential: −0.27 V. Figure S7. The physical picture display of the formaldehyde sensor with three-electrode system.
