# Supplementary material for: Double-Cabin Galvanic Cell-Synthesizing Nanoporous, Flower-like, Pb-Containing Pd–Au Nanoparticles for Nonenzymatic Formaldehyde Sensor
Source: Molecules. 2024 Jun 11;29(12):2772. doi: 10.3390/molecules29122772 (PMC11206501; doi:10.3390/molecules29122772)
Supplement: Supplementary file 1 [file molecules-29-02772-s001.zip › molecules-3011162-supplementary.pdf]

Supporting Information For

Double Cabin Galvanic Cell Synthesizing Nanoporous Flower-like Pb-containing Pd-Au Nanoparticles for Nonenzymatic Formaldehyde Sensor

Zhao Huang [1,2], Zhongsen Tang [1] and Long Chao [1,*]

[1] Hunan Key Laboratory of Biomedical Nanomaterials and Devices, College of Life Sciences and Chemistry, Hunan University of Technology, Zhuzhou 412007, China;
[2] Key Laboratory of Chemical Biology and Traditional Chinese Medicine Research (Ministry of Educa-tion of China), College of Chemistry and Chemical Engineering, Hunan Normal University, Changsha 410081, China;
*Correspondence: E-mail: chaolong4617@163.com

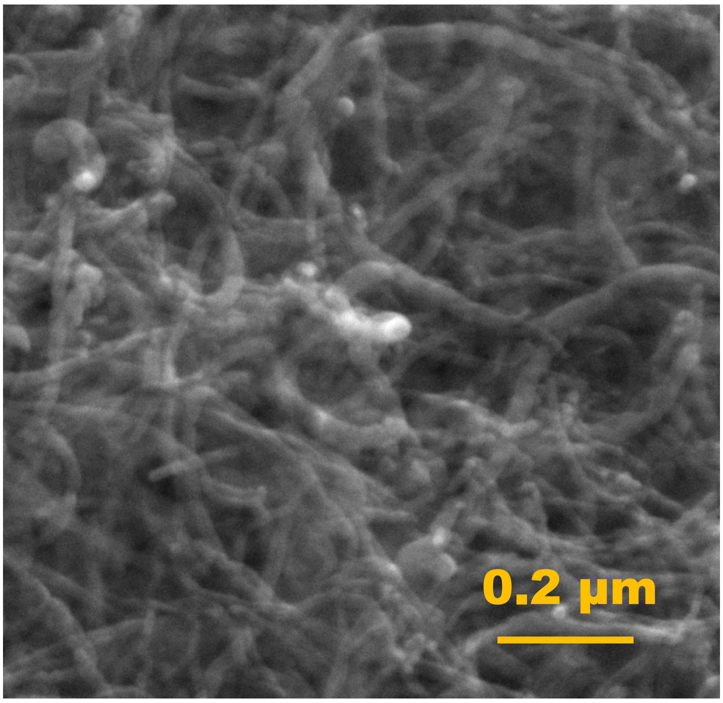

Figure S1. SEM images of MWCNTs/GCE.

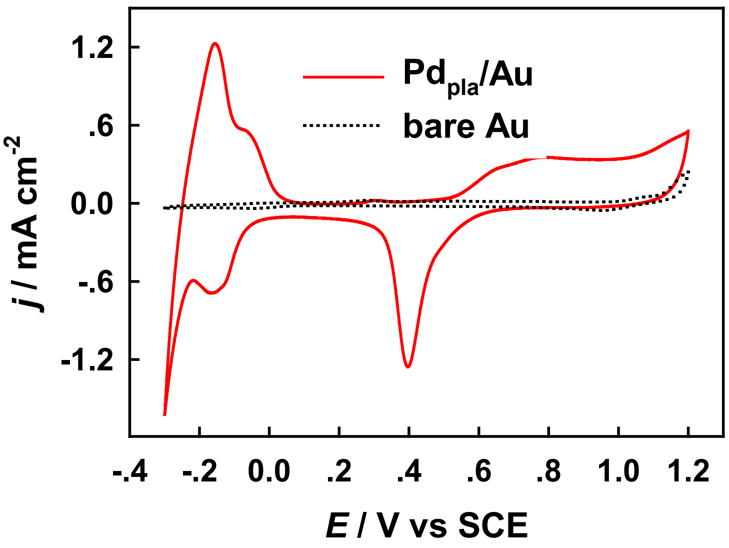

Figure S2. CV curves at Pd$_{pla}$/Au and bare Au electrodes in 0.1 M H_2SO_4 aqueous solution. Scan rate: 50 mV s^{-1}. Pd$_{pla}$/Au electrode was fabricated by electroplating Pd at 0 V vs SCE on a QCM Au electrode in 3.0 mM $PdCl_2$ + 0.1 M $HClO_4$ aqueous solution for 240 s. Here, the reduction peak of Au oxides at ca. 0.85 V disappeared after electroplating Pd on the Au surface, and the big reduction peak of Pd oxides (plus dissolved oxygen) appeared at ca. 0.4 V, indicating that the electrodeposited Pd atoms have fully covered the substrate Au sites. The peaks of hydrogen adsorption/desorption on deposited Pd appeared at ca. 0 ~ −0.22 V.

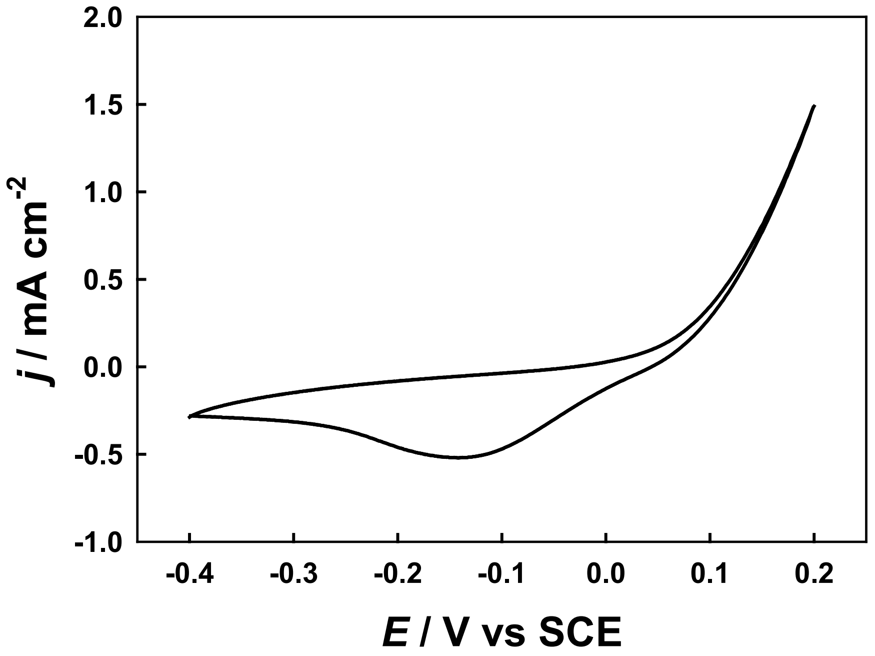

Figure S3. CV curves at Cu plate in 0.1 M $HClO_4$ aqueous solution. Scan rate: 50 mV s^{-1}. Here, during the forward scan, the electrochemical dissolving of bulk Cu occurred form ca. 0.01 V, and the current increased dramatically at more positive potential. During the backward scan, the reduction peak appeared at ca.−0.15 V, corresponding to the reduction of Cu^{2+} to bulk Cu.

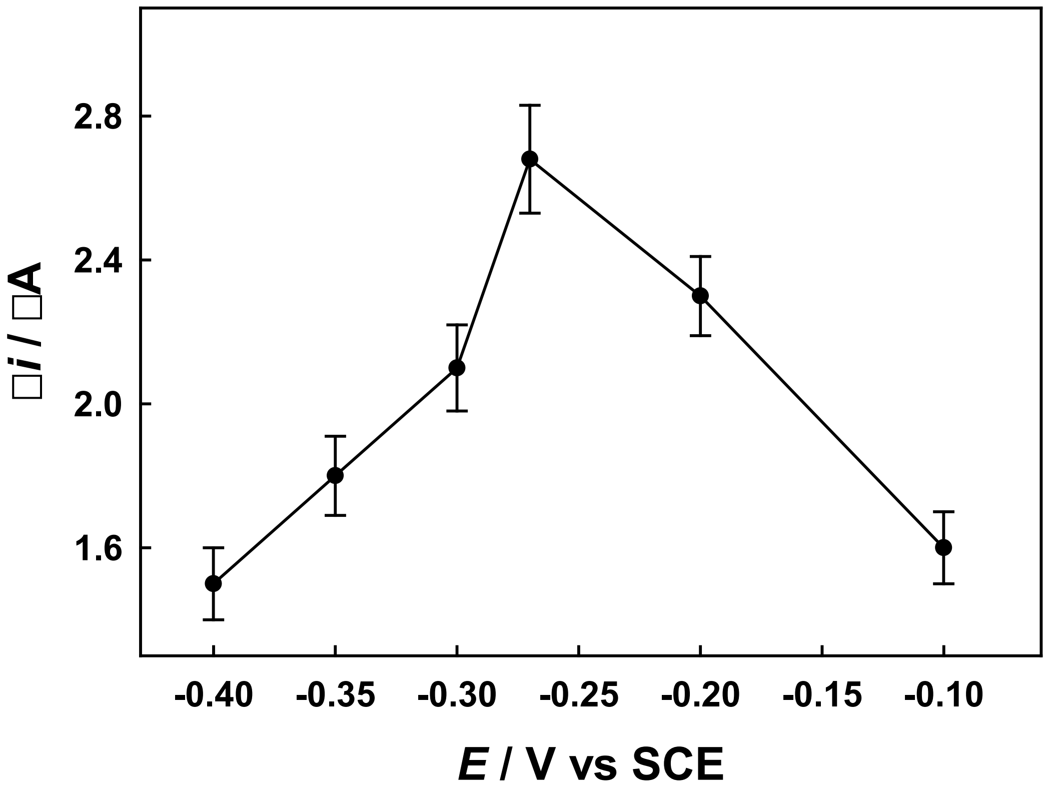

Figure S4. Effects of the applied potential on the amperometric response of 50 μM HCHO for Pb-Pd_3Au_1/MWCNTs/GCE in 0.1 M KOH aqueous solution.

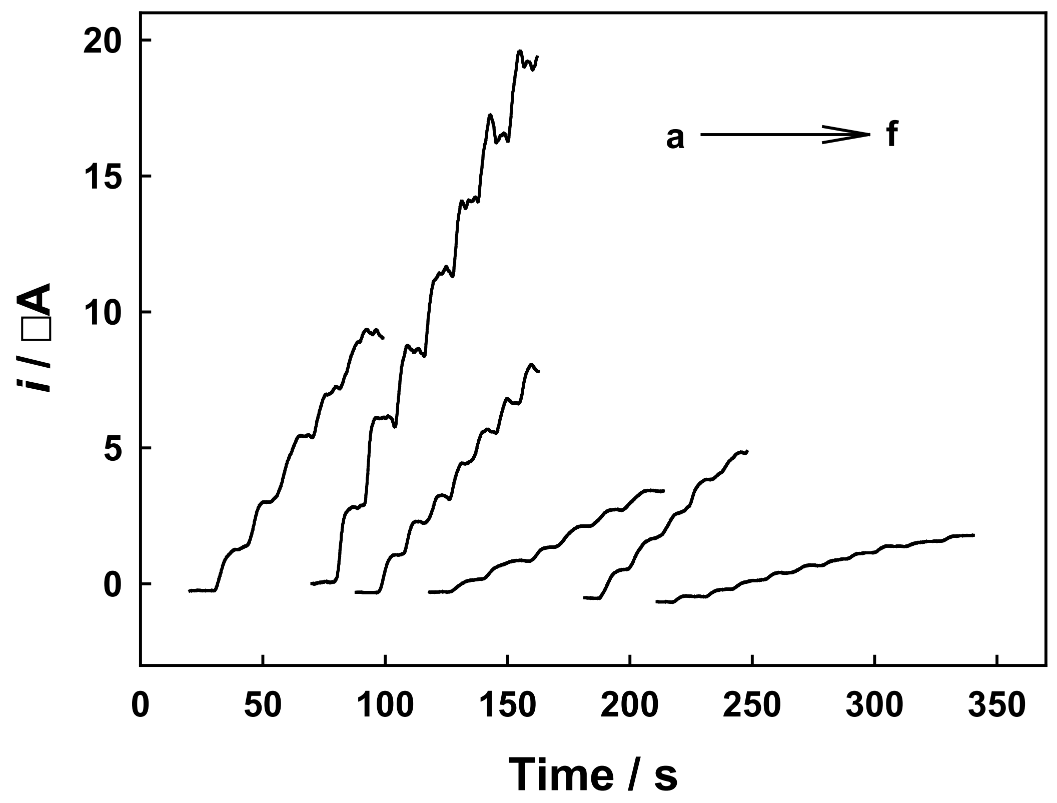

Figure S5. Amperometric response to HCHO at Pb-$Pd_3Au_{0.5}$/MWCNTs/GCE (a), Pb-Pd_3Au_1/MWCNTs/GCE (b), Pb-Pd_3Au_3/MWCNTs/GCE (c), Pb-Pd_3Au_5/MWCNTs/GCE (d), Pd_3Au_1/MWCNTs/GCE (e), Pb-Pd/MWCNTs/GCE (f) upon successive addition of 50 μM HCHO into the stirred 0.1 M KOH aqueous solution. Applied potential: −0.27 V.

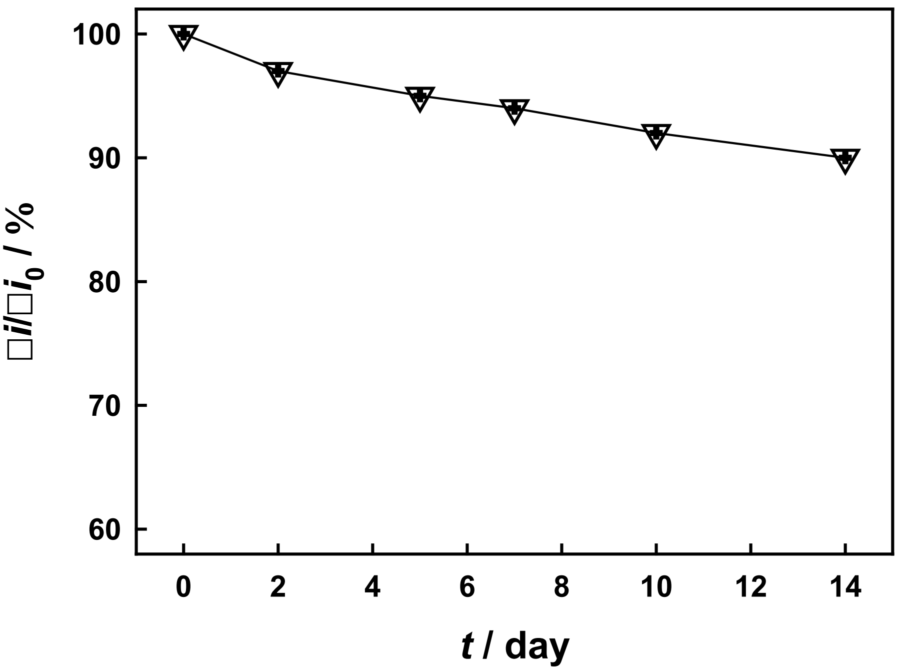

Figure S6. Long-term stability of Pb-Pd_3Au_1/MWCNTs/GCE with repeated detection of 50 μM formaldehyde by potentiostatic method for two weeks. Applied potential: –0.27 V

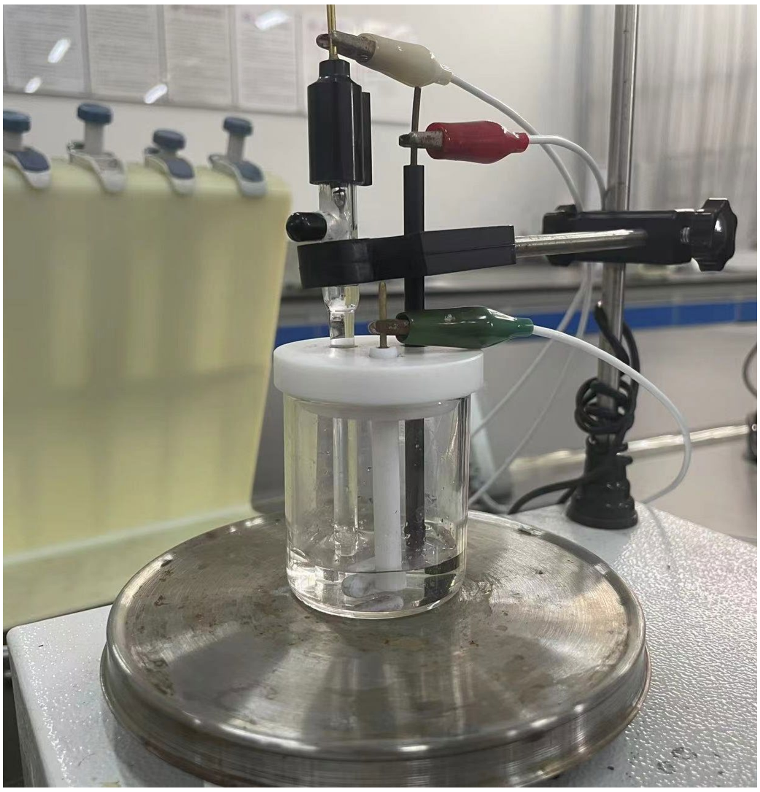

Figure S7. The physical picture display of the formaldehyde sensor with three-electrode system.
